# Gene expression profiling of hematologic malignant cell lines resistant to oncolytic virus treatment

**DOI:** 10.18632/oncotarget.13598

**Published:** 2016-11-25

**Authors:** Nam Hee Lee, Mikyung Kim, Sung Yong Oh, Seong-Geun Kim, Hyuk-Chan Kwon, Tae-Ho Hwang

**Affiliations:** ^1^ SillaJen, Inc., Busan, Korea; ^2^ Department of Physiology, Pusan National University, School of Medicine, Yangsan, Korea; ^3^ Department of Internal Medicine, Dong-A University College of Medicine, Busan, Korea; ^4^ Department of Internal Medicine, Pusan National University Yangsan Hospital, Yangsan, Korea; ^5^ Department of Pharmacology, Pusan National University, School of Medicine, Yangsan, Korea; ^6^ Gene and Cell Therapy Research Center for Vessel-associated Diseases, School of Medicine, Pusan National University, Yangsan, Korea

**Keywords:** oncolytic virus, gene expression profiling, hematologic malignancy

## Abstract

Pexa-Vec (pexastimogene devacirpvec; JX-594) has emerged as an attractive tool in oncolytic virotherapy. Pexa-Vec demonstrates oncolytic and immunotherapeutic mechanisms of action. But the determinants of resistance to Pexa-Vec are mostly unknown. We treated hemoatologic malignant cells with Pexa-Vec and examined the gene-expression pattern of sensitive and resistant cells. Human myeloid malignant cell lines (RPMI-8226, IM-9, K562, THP-1) and lymphoid cancer cell lines (MOLT4, CCRF-CEM, Ramos, U937) were treated with Pexa-Vec. Pexa-Vec was cytotoxic on myeloid cell lines in a dose-dependent manner, and fluorescent imaging and qPCR revealed that Pexa-Vec expression was low in RAMOS than IM-9 after 24 hrs and 48 hrs of infection. Gene expression profiles between two groups were analyzed by microarray. Genes with at least 2-fold increase or decrease in their expression were identified. A total of 660 genes were up-regulated and 776 genes were down-regulated in lymphoid cancer cell lines. The up- and down-regulated genes were categorized into 319 functional gene clusters. We identified the top 10 up-regulated genes in lymphoid cells. Among them three human genes (LEF1, STAMBPL1, and SLFN11) strongly correlated with viral replication. Up-regulation of PVRIG, LPP, CECR1, Arhgef6, IRX3, IGFBP2, CD1d were related to resistant to Pexa-Vec. In conclusion, lymphoid malignant cells are resistant to Pexa-Vec and displayed up-regulated genes associated with resistance to oncolytic viral therapy. These data provide potential targets to overcome resistance, and suggest that molecular assays may be useful in selecting patients for further clinical trials with Pexa-Vec.

## INTRODUCTION

Oncolytic viruses (OVs) mediate tumor regression through selective replication in, and lysis of, tumor cells and induction of systemic anti-tumor immunity without damage to normal cells [1]. Natural or genetically engineered viruses are being investigated for the treatment of solid tumors. There is increasing clinical trials reports supporting their safety and efficacy, both as a monotherapy and in combination with other treatment modalities[2]. However, there was far less attention on hematologic malignancies, may be due to the disseminated nature of leukemia in contrast to discrete masses of solid tumor, inferring that leukemia is less suitable as a target of OVs [3].

Pexastimogene devacirepvec (Pexa-Vec; JX-594) is a cancer specific and transgene inserted oncolytic and immunotherapeutic vaccinia virus engineered to express human granulocyte-macrophage colony-stimulating factor (GM-CSF) and β-galactosidase. Pexa-Vec has multiple mechanisms of action to destroy and eliminate cancer cells [4]. We have demonstrated that Pexa-Vec induces polyclonal antibody-mediated complement-dependent cytotoxicity (CDC) against various malignant cells both in rabbits and in cancer patients [5]. Pexa-Vec has induced objective responses in previous phase 1 or 2 clinical trials [6, 7]. However, no studies have used Pexa-Vec to treat hematologic malignancies.

In this study the oncolytic effects of Pexa-Vec were tested *in vitro* against lymphoid or myeloid cancer cell lines. We also conducted gene expression analysis using a complementary DNA (cDNA) GeneChip microarray to determine the possible predictive gene changes in Pexa-Vec resistant cells compared with sensitive cells. These changes may enable clarify the characteristics of cancers resistant to Pexa-Vec.

## RESULTS

### Vaccinia virus induces cytolysis in myeloid leukemia cell lines, but not in lymphoid leukemia cell lines

**Figure 1:**
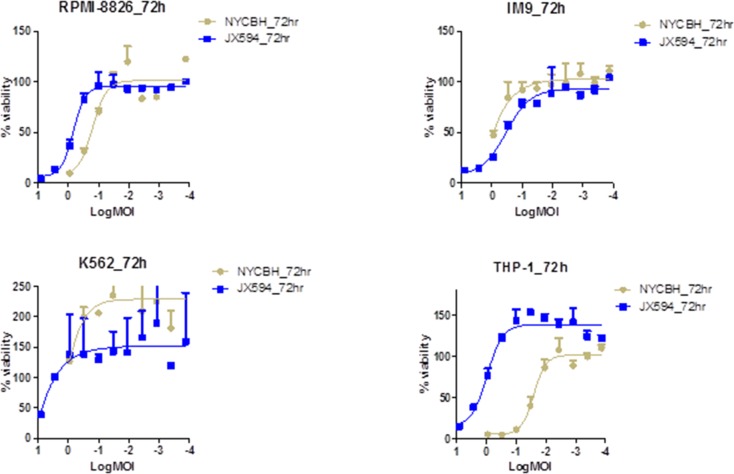
Vaccinia virus induces cytolysis in myeloid leukemia cell lines Percent viability of four myeloid leukemia cell lines at 72 hours of post infection of Pexa-Vec and NYCBH is compared to untreated control. Multiplicity of infection ranges from 8.1 to 0.0001 and represented in log scale. Each assay was tested in triplicate. Error bars = SD.

The viability of four different myeloid leukemia cell lines (RPMI-8826, IM-9, K-562, and THP-1) and lymphoid leukemia cell lines (MOLT-4, CCRF-CEM, Ramos, and U937) were examined 72 hours after treatment with serially diluted vaccinia virus, NYCBH and Pexa-Vec. The cytotoxic effect of vaccinia virus on the myeloid leukemia cell lines was increased in a dose-dependent manner for both viruses, with THP-1 cells more sensitive to NYCBH strain than Pexa-Vec (Figure 1). Vaccinia virus ED_50_ doses after viral treatment on myeloid cell lines were calculated and THP-1 cells confirmed to be the most sensitive to NYCBH and IM-9 cells were the most to Pexa-Vec. Unlike other myeloid cell lines examined, the viability of NYCBH infected THP-1 was significantly decreased compare to Pexa-Vec because Pexa-Vec was genetically attenuated virus by disrupting thymidine kinase region of the wild type virus. This cytolysis effect on myeloid cells was relatively resistant compare to ED_50_ values of vaccinia infected solid tumors including colon, prostate, breast, ovarian, lung, kidney and etc. The cytopathic effect of NYCBH on K-592 cells was not evident within the range of diluted virus examined. However, it was expected that a cytotoxic effect would be present at an infection with virus at a MOI higher than 10, judging from the increase of inhibitory effect on the leukemia cell growth in accordance with the increase of the concentration of treated virus. Furthermore, myeloid cell line K562 proliferates more than control under low MOI of viral infection. This phenomenon can be explained by the alteration of cell cycle progression. Virus infection has a considerable impact on the physiology and metabolism of the host cell and low MOI of viral infection stimulates cell proliferation. When vaccinia virus infects, the percentage of cells in G1 decreases and S phase cells get increased. The degree of cell growth differs by cell and virus type and time of incubation.

**Figure 2:**
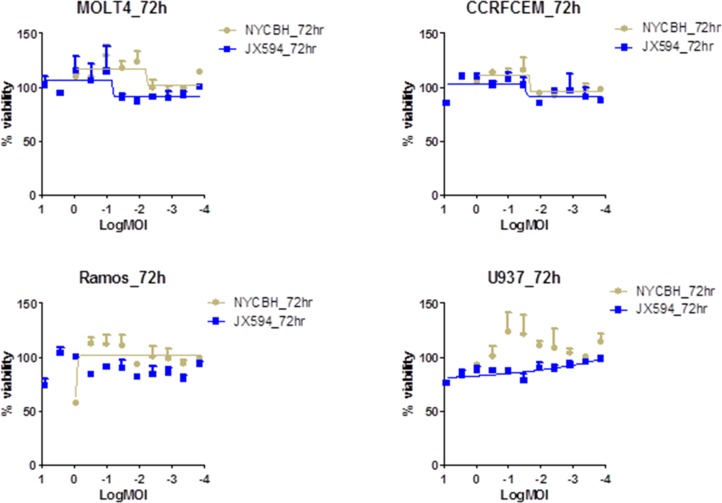
Lymphoid cell lines are resistance to the vaccinia virus infection Percent viability of four lymphoid cell lines at 72 hours of post infection of Pexa-Vec and NYCBH shows their resistance to viral infection. Multiplicity of infection ranges from 8.1 to 0.0001 and represented in log scale. Each assay was tested in triplicate. Error bars = SD.

In contrast, all of lymphoid leukemia cell lines investigated were resistant to NYCBH and Pexa-Vec infection (Figure 2). Lymphoid leukemia cells were not killed and their growth was not inhibited by all virus dilutions, with a similar cell growth to control. A cytopathic effect was not prominent even at the highest concentration of infected viruses of 10 MOI. The similar findings with the lymphoid cell lines to both viruses suggest that lymphoid leukemia cells are resistant to oncolytic vaccinia virus infection. The ED_50_ values for lymphoid cell lines were ambiguous since cells were not killed within the examined range of MOI (Figure 2).

### Microscopic images of leukemia cell lines after viral infection shows different effect of oncolytic virus on the cell growth of myeloid and lymphoid cell lines

**Figure 3:**
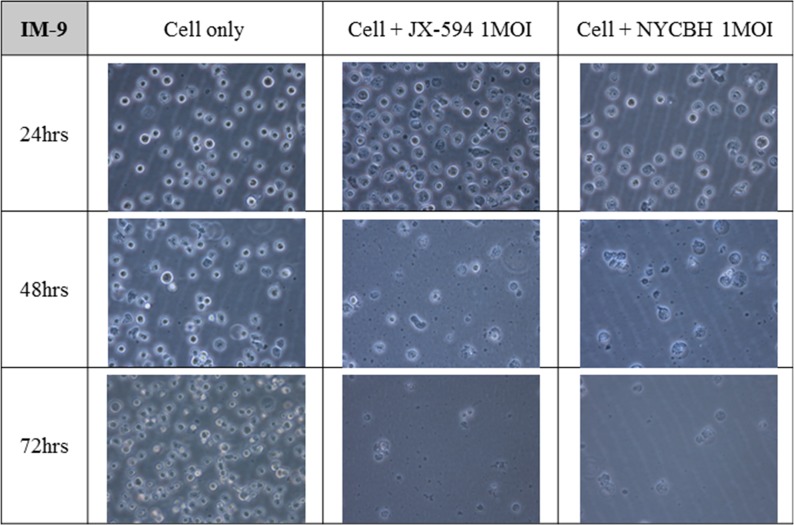
Microscopic images of myeloid leukemia cell line over time following viral infection The myeloid cell line IM-9 was infected with 1 MOI of Pexa-Vec or NYCBH and incubated for 72 hours. The representative microscopic images were captured every 24 hours post-infection. Mock infected cells were used as control.

**Figure 4:**
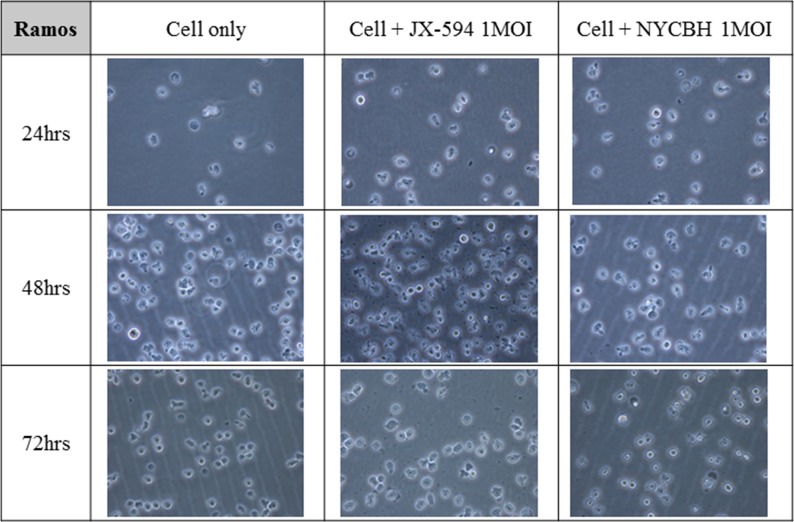
Microscopic images of lymphoid leukemia cell line over time following viral infection The lymphoid cell line Ramos was infected with 1 MOI of Pexa-Vec or NYCBH and incubated for 72 hours and the representative microscopic images were captured at every 24 hours post-infection. Mock infected cells were used as control.

Figures 3 and 4 depict microscopic details of IM-9 myeloid leukemia cells and Ramos lymphoid leukemia cells at 24, 48 and 72 hours post-infection with Pexa-Vec or NYCBH (MOI of 1). A time-dependent cytopathic effect was readily evident in the virus-treated group compared to IM-9 mock control. When Pexa-Vec was used to infect Ramos cells, the change of the cells was similar to control. A cytotoxic effect for Ramos cells was not apparent until 72 hours post-infection for NYCBH, in contrast with IM-9 cells. This result again revealed that oncolytic vaccinia virus does infect well on myeloid leukemia cells but it does not show antitumor effect on lymphoid leukemia cells.

### Vaccinia virus replicates in myeloid leukemia cells, but not lymphoid leukemia cells

**Figure 5:**
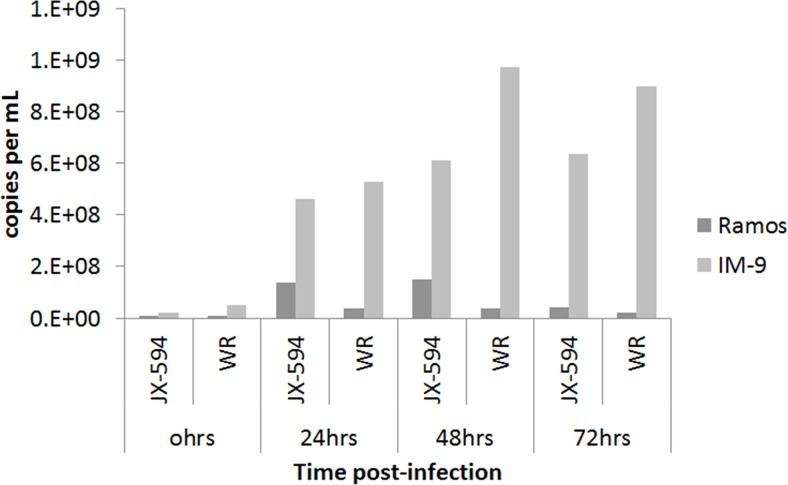
Physical viral particle count by qPCR assay The number of physical DNA copies targeting E9L gene of vaccinia virus is plotted against time after viral infection on IM-9 and Ramos cell lines. The total DNA was isolated from infected cell harvest using QIAamp DNA blood mini kit. Data is shown on average of duplicate runs. JX-594:Pexa-Vec, WR: western reserve virus.

The number of physical viral particles in IM-9 and Ramos cells harvested 24, 48 and 72 hours after Pexa-Vec or Western Reserve vaccinia virus infection was examined by qPCR assay (Figure 5). The number of viral particles in IM-9 cells increased in a time-dependent manner after virus infection, demonstrating the replication of the vaccinia virus. The relatively greater replication of the Western Reserve virus compared to the Pexa-Vec virus is likely due to the intentionally disrupted thymidine kinase gene, since its translational product is required for the DNA synthesis. Malfunction of the gene would make replication capability of Pexa-Vec inferior to the wild type virus with an intact gene. In case of Western Reserve virus, the number of viral particles was maximum at 48 hours post-infection, with no increase at 72 hours. This observation likely reflects the lack of host IM-9 cells for viral replication due to the simultaneous virus-mediated killing of the host cells. The increase in viral particle number was not remarkable and time-dependent in Ramos cells infected with Pexa-Vec or Western Reserve virus. This finding indicates that the viruses did not replicate and amplify in Ramos cells, which was expected based on the cytotoxicity assay results and microscopic observation. Oncolytic vaccinia virus displayed antitumor activity in myeloid leukemia cells like it attacks solid tumor cells by replicating inside the target cells. But, the lack of an oncolytic effect on lymphoid leukemia cells indicates that vaccinia virus cannot infect and/or replicate well in lymphoid leukemia cells.

**Figure 6:**
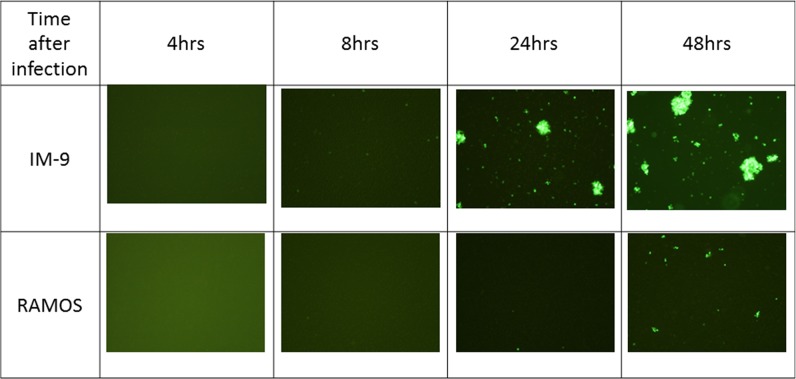
Fluorescence images after viral infection on leukemia cell lines The GFP expression was visualized by fluorescence microscopy at 4, 8, 24 and 48 hours post-infection on IM-9 and Ramos cell lines. GFP fluorescence imaging showed that the virus infected on myeloid cell line IM-9 was replicated successfully after 24 hours. The representative images were shown here.

GFP expression in IM-9 and Ramos cells was visualized by fluorescence microscopy at 4, 8, 24 and 48 hours post-infection with vaccinia virus (Figure 6). The expression of GFP protein inside the cells was clearly seen at 24 hours and the expression was increased at 48 hours in IM-9 cells, corroborating the qPCR results of successful infection of IM-9 cells. GFP expression was also observed at 48 hours post-infection in Ramos cells but was markedly less than the expression in IM-9 cells, indicating the inefficient (almost negligible) infection of Ramos cells by vaccinia virus.

### Gene expression differs in myeloid and lymphoid malignant cells

To begin to understand the genetic differences between Pexa-Vec sensitive and Pexa-Vec resistant hematologic malignant cells, the RNA expression of sensitive myeloid and resistant lymphoid cells were analyzed using microarray analysis. This analysis allowed us to detect differences in cell function and pathways between Pexa-Vec sensitive and Pexa-Vec resistant cell lines, and identify candidate genes to determine Pexa-Vec sensitivity.

**Figure 7:**
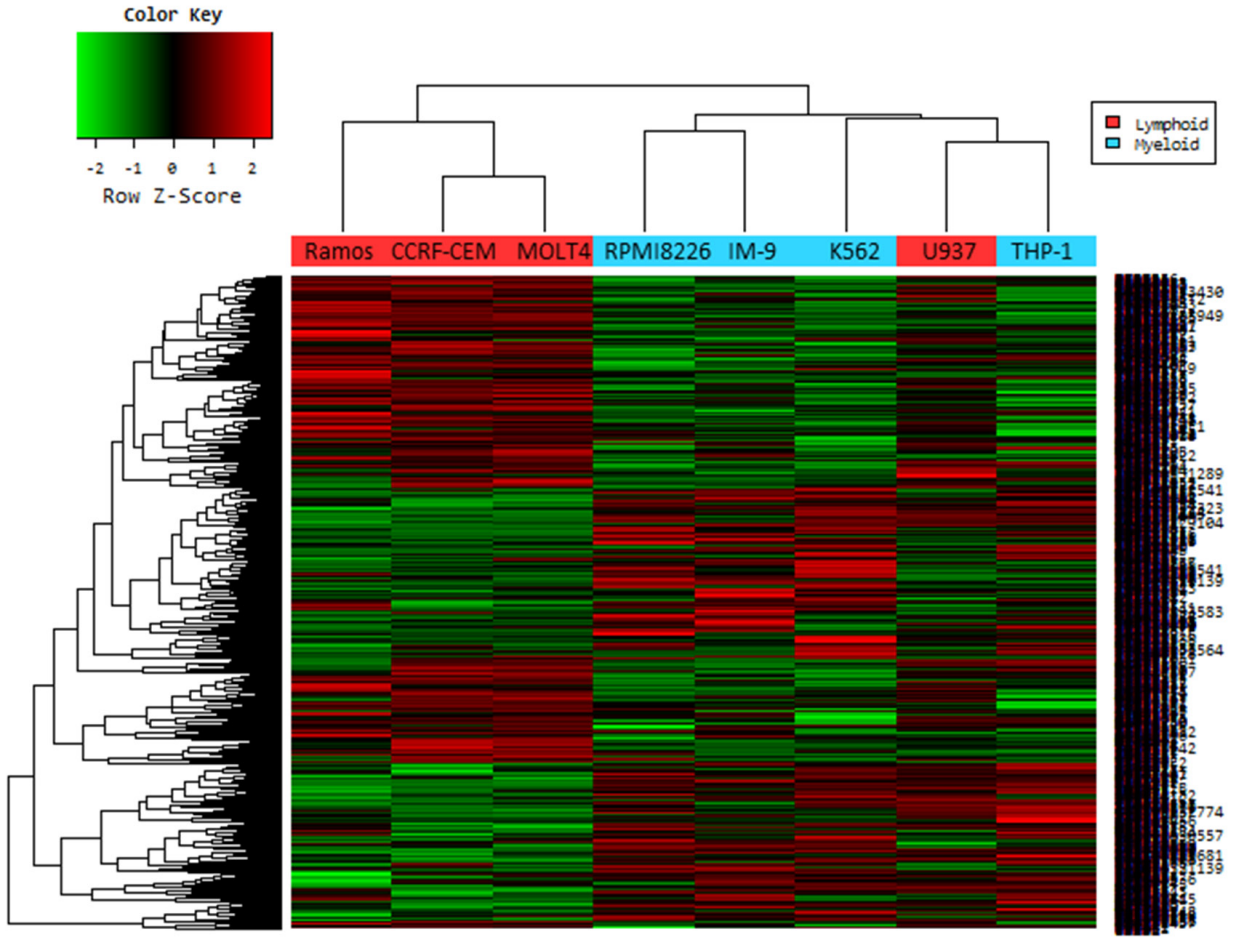
Hierarchical cluster analysis of microarray on virus infected leukemia cell lines In a microarray raw data set, cell lines are ordered in columns and gene expression value are given in rows. The columns labeled in red (Ramos, CCRF-CEM, MOLT-4 and U937) are data from the lymphoid cell lines infected with Pexa-vec, and in blue (RPMI-8226, IM-9, K562 and THP-1) are from myeloid cell lines. Red cells indicate high expression and green cells indicate low expression

Genes with at least 2-fold increase or decrease in their expression were identified. A total of 660 genes were up-regulated and 776 genes were down-regulated in the lymphoid cell lines. Changes were especially dramatic in the case of up-regulated genes: more than 50 genes were induced 5-fold or higher and another 150 genes were expressed 3- to 4-fold of control cells. Hierarchical clustering revealed the similarity among the commonly changed genes (Figure 7). Only one lymphoid cell line (U937; histiocytic lymphoma) was misclassified.

Using the DAVID functional annotation clustering tool, the up- and down-regulated genes were categorized into 319 functional gene clusters. Genes that could be used to identify cell lines resistant to Pexa-Vec were grouped into functional categories. Genes related to biologic processes, cellular components, and molecular functions are presented in the Supplementary Figure 1, 2, and 3, respectively. The top seven significant molecular and cellular function groups (according to P-value) included phosphoprotein (507 genes, 47.4%, P = 2.61E-15), mutagenesis site (162 genes, 15.1%, P = 1.86E-07), regulation of programmed cell death (80 genes, 7.5%, P = 8.55E-07), regulation of cell death (80 genes, 7.5%, P = 9.95E-07), lysosome (25 genes, 2.3%, P = 1.10E-06), regulation of apoptosis (79 genes, 7.4%, P = 1.14E-06), and surface antigen (13 genes, 1.2%, 1.19E-06).

Ten highly up-regulated and 10 highly down-regulated genes of interest are listed in Table 1. The top 10 up-regulated genes in lymphoid malignant cells resistant to Pexa-Vec were identified. The highest change was found in the gene named LEF1 (lymphoid enhancer-binding factor 1) where fold change was 12.1. The fold change values for each gene were calculated and specified in Table 1. Among them, three human genes strongly correlated with viral replication: LEF1, STAMBPL1 (STAM binding protein-like 1), and SLFN11 (schlafen family member 11). Up- regulation of PVRIG (poliovirus receptor related immunoglobulin domain containing), LPP (LIM domain containing preferred translocation partner in lipoma), CECR1 (cat eye syndrome chromosome region, candidate 1), Arhgef6 (Rac/Cdc42 guanine nucleotide exchange factor [GEF) 6), IRX3 (iroquois homeobox 3), IGFBP2 (insulin-like growth factor binding protein 2), and CD1d were found. All these genes have unknown functions in viral replication or infection.

**Table 1:** Top 10 genes up- and down-regulated in Pexa-Vec resistant hematologic cancer cell lines

Reference seq_NM	Gene Symbol	Gene ID	fold change	p value	Definition
NM_016269.2	LEF1	51176	12.1	4.10E-07	Homo sapiens lymphoid enhancer-binding factor 1 (LEF1), mRNA.
NM_024070.3	PVRIG	79037	11.2	1.76E-05	Homo sapiens poliovirus receptor related immunoglobulin domain containing (PVRIG), mRNA.
NM_152270.2	SLFN11	91607	10.0	9.94E-09	Homo sapiens schlafen family member 11 (SLFN11), mRNA.
NM_005578.2	LPP	4026	9.7	0.009	Homo sapiens LIM domain containing preferred translocation partner in lipoma (LPP), mRNA.
NM_177405.1	CECR1	51816	9.3	0.024	Homo sapiens cat eye syndrome chromosome region, candidate 1 (CECR1), transcript variant 2, mRNA.
NM_004840.2	ARHGEF6	9459	8.9	0.024	Homo sapiens Rac/Cdc42 guanine nucleotide exchange factor (GEF) 6 (ARHGEF6), mRNA.
NM_024336.1	IRX3	79191	8.5	1.07E-08	Homo sapiens iroquois homeobox 3 (IRX3), mRNA.
NM_020799.2	STAMBPL1	57559	7.7	0.026	Homo sapiens STAM binding protein-like 1 (STAMBPL1), mRNA.
NM_000597.2	IGFBP2	3485	6.7	0.033	Homo sapiens insulin-like growth factor binding protein 2, 36kDa (IGFBP2), mRNA.
NM_001766.3	CD1D	912	6.7	0.033	Homo sapiens CD1d molecule (CD1D), mRNA.
NM_139030.3	CD151	977	−9.9	0.001	Homo sapiens CD151 molecule (Raph blood group) (CD151), transcript variant 2, mRNA.
NM_001620.1	AHNAK	79026	−10.4	0.000	Homo sapiens AHNAK nucleoprotein (AHNAK), transcript variant 1, mRNA.
NM_003302.2	TRIP6	7205	−10.5	0.015	Homo sapiens thyroid hormone receptor interactor 6 (TRIP6), mRNA.
NM_002305.3	LGALS1	3956	−10.8	0.001	Homo sapiens lectin, galactoside-binding, soluble, 1 (LGALS1), mRNA.
NM_145792.1	MGST1	4257	−11.1	0.001	Homo sapiens microsomal glutathione S-transferase 1 (MGST1), transcript variant 1a, mRNA.
NM_002727.2	SRGN	5552	−12.4	1.54E-06	Homo sapiens serglycin (SRGN), mRNA.
NM_001759.2	CCND2	894	−13.0	2.91E-08	Homo sapiens cyclin D2 (CCND2), mRNA.
NM_174908.2	CCDC50	152137	−13.0	6.91E-05	Homo sapiens coiled-coil domain containing 50 (CCDC50), transcript variant 1, mRNA.
NM_000889.1	ITGB7	3695	−13.7	0.0002	Homo sapiens integrin, beta 7 (ITGB7), mRNA.
NM_020992.2	PDLIM1	9124	−15.3	0.0001	Homo sapiens PDZ and LIM domain 1 (PDLIM1), mRNA.

## DISCUSSION

Since the approval of the first oncolytic virus in China [8], there is unmet medical needs using oncolytic vaccinia viruses that target hematological malignancies. Several oncolytic viruses are in development, including coxsackievirus A21 for multiple myeloma [9], reovirus for lymphoma [10], and myxoma for acute myeloid leukemia (AML) [11]. Vaccine-strain measles and mumps virus combinations has also led to a synergistic increase in cytotoxicity in myeloid leukemia cells [12].

Pexa-Vec is an oncolytic and immunotherapeutic virus and it appears to replicate and kill cancer cells but not normal human cells [4]. We investigated the direct effect of Pexa-Vec on hematologic malignant cells, and showed that Pexa-Vec can replicate and induce death in cell lines derived from both myeloid leukemia and multiple myeloma origin, not of lymphoid origin. These findings support the potential utility of Pexa-Vec for myeloid leukemia or multiple myeloma as first-line treatment, salvage therapy, or purging prior to autologous stem cell transplantation. Furthermore, these results support the incorporation of Pexa-Vec into the design of future clinical trials for the treatment of myeloid malignancy.

A recent report described that human mesenchymal stromal cells can deliver oncolytic measles virus into acute lymphoblastic leukemia (ALL) cells, even though in the presence of anti-measles virus antibodies [13]. There is increasing preclinical data that the efficacy of OVs may be increased by a combination with other anti-cancer drugs, as has been shown for reovirus enhanced rituximab mediated antibody dependent cellular cytotoxicity against chronic lymphocytic leukemia [14]. Considering these results, Pexa-Vec may potentially be used to treat lymphoid malignancies with other modalities to overcome the resistance or improve the outcome.

Gene expression profiling is a powerful technology for the diagnosis of subtypes of hematologic malignancies with high accuracy. International microarray innovations in leukemia study group reported the clinical usefulness of microarray-based gene expression profiling in the diagnosis and subclassification of leukemia [15]. The authors reported that the stage I study achieved 92.2% classification accuracy and median specificity of 99.7%. In stage 2, 95.6% median sensitivity and 99.8% median specificity were shown. Gene-expression patterns in drug-resistant ALL cells and response to treatment was also reported[16]. They found differentially expressed genes in ALL patients, that were sensitive or resistant to predinisolone, vincristine, asparaginase, or daunorubicin. Therefore, it is possible that we can diagnosis types of leukemia, and predict chemoresponse to ALL by using gene expression profiling.

In order to clarify potential mechanisms involved in Pexa-Vec resistance, gene-expression patterns comparing sensitive myeloid malignant cells with resistant lymphoid cancer cells were examined via microarray analysis after total RNA was extracted from each cell group. The cDNA microarray GeneChip technique is commonly used for genome-wide expression profiling of cellular responses to many external environmental stimuli [17, 18]. For these experiments, we used the Illumina Human HT-12 v4.0 Expression Beadchip, which contains almost 47,322 probe sets. We selected filtered data and found 1,336 probes with a fail count rate less than 0.05 and more than a 2-fold change between sensitive and resistant of cancer cells toward Pexa-Vec. When the data were analyzed using DAVID clustering, these genes comprised mostly enzymes, transporters, and transcription regulators. Their most statistically significant roles were in immune response, inflammation and death signaling, cell morphology, cellular movement, cellular growth and proliferation, and cell-to-cell signaling and interaction.

Several reports have described genes related to OVs resistance. In one, a mechanism identified in cancer cells to resist infection by herpes viruses was decreased FN1 expression, which may have reduce viral attachment [17]. Another study revealed the immunoglobulin-like transcript 2 (ILT2) gene as a marker of regulation of CD4+ and suppressor CD8+ T cell responses. Down regulation of ILT2 gene was predictive marker of clinical responses in malignant melanoma patients treated with vaccina-B7.1 [18]. There was another report that inhibition of virus endocytosis and intact interferon-mediated defenses are responsible for M protein mutant vesicular stomatitis virus resistance in pancreatic cancer cells [19]. We identified top 10 up-regulated genes in lymphoid cells. Among them, LEF1, STAMBPL1, and SLFN11 human genes strongly correlated with viral replication. As well, up-regulation of PVRIG, LPP, CECR1, Arhgef6, IRX3, IGFBP2, and CD1d were related to Pexa-Vec resistance. These seven genes have not been previously reported from other studies, perhaps because of the use of different cell lines, treated OVs, and the microarray chips used [17–19].

LEF1 is a major mediator of Wnt signaling. It binds to β-catenin to activate the downstream cascade [20]. Increased expression of LEF1 is related to expression of cell cycle and growth-promoting genes, and disturbs differentiation in hematopoiesis [21]. Most immature T lymphocyte have high levels of LEF-1 expression, whereas in normal, non-transformed mature T lymphocytes there are only low levels of LEF-1 gene [22]. In one report high LEF1 expression and mutation was associated with high-risk of leukemia and LEF1 high expression or mutations were related with leukemogenesis of ALL [23]. Other report showed that T cell factor 1 and LEF1 suppress human T-cell leukemia virus type-1 replication through inhibiting Tax-dependent viral expression and activation of nuclear factor-kappa B [24].

STAMBPL1 (STAM binding protein-like 1) is a JAMM family member, it works as a metalloprotease with specificity for K63-linked polyubiquitination chains [25]. STAMBPL1 is also a positive regulator of Tax activation of NF-κB. STAMBPL1 is essential deubiquitinating enzyme for the export of Tax from the nucleus to the cytoplasm, where it triggers IKK and NF-κB activation in human T-cell leukemia virus type-1 [26]. As a result, viral transcription and replication are greatly suppressed by either LEF1 or STAMBPL1, resulting in selective viral replication in LEF1/STAMPBPL1 low-expressing cells.

Human SLFN11 was known to break the production of human immunodeficiency virus (HIV). SLFN11 specifically inhibits viral protein synthesis at the late stage of virus production in HIV infected cells in a codon-usage-dependent manner [27]. Expression of SLFN 11 is significantly elevated in CD4+ T cells from well controllers as compared to poor-controllers, showing SNFL11 to be an antiviral factor [28]. However, little is known about SLFN11 functions in cancer cells. There was a report that SLFN11 enhances sensitivity to DNA damaging agents but not to other chemotherapeutic drugs, suggesting that SLFN11 participates in the DNA damage response [29]. We propose that the up-regulation of LEF1, STAMBPL1, and SLFN11 during viral replication may be used as a biomarker of Pexa-Vec treatment.

CD1d-restricted T-cell populations have a role in immune surveillance, which is mediated via the maturation of antigen-presenting cells and IL-12 induction through Natural Killer (NK) and CD8^+^ T cells [30]. Although vaccinia virus infection activates and mobilizes a lot of lymphocyte populations, the virus blocks CD1d-mediated antigen presentation to NKT cells although cellular CD1d expression was not changed by vaccinia virus [31].

LPP (LIM domain containing preferred translocation partner in lipoma) can regulate trafficking of signaling proteins from nucleus to cytoplasm. LPP is associated with cell migration, proliferation, and transcription [32]. LPP was overexpressed in lung carcinoma, soft tissue sarcoma, and leukemia [33]. However, the function of LPP remains unknown. More data are needed to reveal the physiological role of LPP and to clalify the functional differences between normal and altered LPP signaling.

IRX3 is a member of the Iroquois homeobox gene family. It works an early step of neural development [34]. Analizing data of the transcriptional profiles of human colorectal adenoma samples showed IRX3 as one of the most up-regulated transcription factors compared to normal tissue [35]. IRX protein prohibits tumor cells to respond to TGF‐β during the transition from adenoma to carcinoma in the human colon [36].

IGFBP2 (insulin-like growth factor binding protein 2) has been reported as a potential biomarker in ALL [37]. Serum IGFBP-2 levels in ALL patients were significantly higher than those in the control group at diagnosis, but returned to normal value after intensive chemotherapy. However, the role of IGFBP2 in cancer is unclear. In general, IGFBP2 is related to oncogenesis and its expression is often elevated in cancer. But, there are several conflicting reports that IGFBP2 acts in a tumor suppressor role [38].

Arhgef6 (aka alpha-PIX or Cool-2) is a Rac1/Cdc42-specific guanine nucleotide exchange factor binds to β-parvin/affixin and Calpain-4, and makes a complex that co-localizes with integrin-linked kinase in migrating cells [39]. Arhgef6 has been involved in the formation of focal adhesion structures essential for cell motility. Recently, the up-regulation of Arhgef6 in human medulloblastomas, and its participation in experimental medulloblastomagensis was reported [40].

CECR1 (cat eye syndrome chromosome region, candidate 1) encoding the ADA2 (adenosine deaminase2) protein. It has been shown that ADA2 level is elevated in sera from HIV-infected patients, suggesting that ADA2 activity is one of biomarkers to improve the diagnosis and follow-up treatment of HIV infection[41]. There was a report that ADA2 is secreted by monocytes undergoing differentiation into macrophages or dendrite cells, and stimulates macrophage proliferation, meaning ADA2 does unique roles in cell signaling in the human immune system [42].

## MATERIALS AND METHODS

### Viruses

Pexa-Vec is a Wyeth strain vaccinia virus engineered for viral thymidine kinase (TK) gene inactivation, and expression of the human granulocyte-macrophage colony stimulating factor (hGM-CSF) and β-galactosidase (β-gal) transgenes under control of the synthetic early-late and p7.5 promoters, respectively. JX-594-GM-CSF-GFP has been genetically manipulated to encode for hGM-CSF and green fluorescent protein in the disrupted thymidine kinase locus. The vaccinia virus Wyeth strain and NYCBH were obtained from American Type Culture Collection.

### Cell lines

RPMI-8226 and IM-9 [human multiple myeloma (MM); Korean Cell Line Bank (KCLB)], K562 [human chronic myelogenous leukemia (CML); KCLB], and THP-1 [acute monocytic leukemia (AMOL); KCLB] were cultured in RPMI 1640 (HyClone, Logan, UT, USA) supplemented with 10% fetal bovine serum (FBS; HyClone) and penicillin and streptomycin (HyClone). MOLT4 and CCRF-CEM [human acute lymphoblastic leukemia (ALL); KCLB] cells, Ramos (human Burkitt's lymphoma; KCLB) cells, and U937 (human histiocytic lymphoma; KCLB) cells were separately cultured in RPMI 1640 supplemented with 10% FBS, penicillin and streptomycin.

### Cell cytotoxicity assay

Cytotoxicity of the virus was assessed 3 days post-infection (p.i.) using Cell Counting Kit-8 (Dojindo Molecular Technologies, Rockville, MD, USA) according to the manufacturer's instructions. Cell viability was determined by measuring absorbance at 490 nm using a Synergy H1 96-well plate absorbance reader (BioTek Instruments, Winooski, VT, USA). A dose-response curve was created by nonlinear regression, allowing determination of a 50% effective concentration (EC50; viral dose required to kill 50% of the cells). Each assay was conducted in triplicate.

### JX-594-GFP imaging

IM-9 and Ramos cells were plated at 4e5 cells/well and cultured overnight. Cells were then infected with JX-594-GFP at a multiplicity of infection (MOI) of 1.0 and cultured in the presence of serum for 24 and 48 hours. During culture, the cells were monitored for susceptibility to viral gene expression evident as the production of GFP by fluorescence microscopy.

### Quantitative PCR (qPCR)

The concentration of the Pexa-Vec genome in cell lysates over time was determined using qPCR. Samples were collected 0, 24, 48, and 72 hours following infection. Total DNA was isolated using QIAamp DNA Blood mini Kit (Qiagen, Hilden, Germany) and quantified using a model ND-1000 spectrophotometer (Nano Drop Technologies, Wilmington, DE, USA). The copy numbers of genome were quantified using a primer set specific for 9EL gene (E9L-F1880 5′-GAA CAT TTT TGG CAG AGA GAG CC-3′ E9L-R2057 5′-CAA CTC TTA GCC GAA GCG TAT GAG-3′ E9L-p1924S-MGB 6'FAM-CAG GCT ACC AGT TCA A-MGBNFQ-3′) and 10 or 100 ng of DNA template using an ABI 7300 real time PCR machine (Applied Biosystems, Franklin Lakes, NJ, USA).

### RNA extraction

Total RNA was extracted using Trizol reagent (Invitrogen, Carlsbad, CA, USA) according to the manufacturers' protocol. RNA purity and integrity were evaluated using a model ND-1000 spectrophotometer (Nano Drop Technologies) and model 2100 bioanalyzer (Agilent Technologies, Palo Alto, CA, USA).

### Labeling and purification

Total RNA was amplified and purified using the TargetAmp-Nano labeling kit for Illumina Expression BeadChip (EPICENTRE, Madison, WI, USA) to yield biotinylated cRNA according to the manufacturer's instructions. Briefly, 500 ng of total RNA was reverse-transcribed to cDNA using a T7 oligo (dT) primer. Second-strand cDNA was synthesized, in vitro transcribed, and labeled with biotin-NTP. After purification, the cRNA was quantified using the aforementioned ND-1000 spectrophotometer.

### Hybridization and data export

Labeled cRNA samples (75 ng) were hybridized to each Human HT-12 v4.0 Expression Beadchip for 17 hours at 58°C, according to the manufacturer's instructions (Illumina, Inc., San Diego, CA, USA). Detection of array signal was carried out using fluorolink streptavidin-Cy3 (GE Healthcare Bio-Sciences, Little Chalfont, UK) following the bead array manual. Arrays were scanned with a bead array reader confocal scanner according to the manufacturer's instructions (Illumina, Inc.).

### Raw data preparation and statistical analyses

The quality of hybridization and overall chip performance were monitored by visual inspection of both internal quality control checks and the raw scanned data. Raw data were extracted using the software provided by the manufacturer (Genome Studio v2011.1 and Gene Expression Module v1.9.0; Illumina, Inc.). Array probes were logarithm transformed and normalized by the quantile method. Statistical significance of the expression data was determined using fold-change. To control for multiple testing, the false discovery rate (FDR) method was used, with a cutoff of 0.05. For a differentially expressed genes (DEG) set, hierarchical cluster analysis was performed using complete linkage and Euclidean distance as a measure of similarity. Expression of specific genes was determined from raw microarray data. Gene expression data were normalized and the absolute fold-change expression was determined. At least a 2-fold increase or decrease in expression was considered to be significant using unpaired T-probe with Benjamini-Hochberg correction. Gene-enrichment and functional annotation analysis for significant probe list was performed using DAVID (http://david.abcc.ncifcrf.gov/home.jsp). All data analyses and visualization of differentially expressed genes were conducted using R 3.0.2 (www.r-project.org). All statistical analyses were performed using Prism 5.0 software (GraphPad Software, La Jolla, CA, USA). The unpaired t-test was used to assess differences.

## CONCLUSIONS

Myeloid cancer cells were sensitive to Pexa-Vec and the gene expression profile of lymphoid malignant cells rendered them resistant to Pexa-Vec, which has not been previously reported. Further investigation of the mechanisms associated with the emergence of Pexa-Vec resistance is needed to develop strategies to overcome this potential limitation. The identified genes may also be studied concerning their value in patient selection criteria for clinical trials of Pexa-Vec. Furthermore, the present findings could help identify possible biomarkers that predict response to Pexa-Vec treatment. Our study provides a framework for the observation of possible cellular events, as well as potential biologic and molecular targets, to overcome Pexa-Vec resistance.

## SUPPLEMENTARY FIGURES


