# Supplementary material for: Gene expression profiling of hematologic malignant cell lines resistant to oncolytic virus treatment
Source: Oncotarget. 2016 Nov 25;8(1):1213–25. doi: 10.18632/oncotarget.13598 (PMC5352049; doi:10.18632/oncotarget.13598)
Supplement: Supplementary file 1 [file oncotarget-08-1213-s001.pdf]

# Gene expression profiling of hematologic malignant cell lines resistant to oncolytic virus treatment

## SUPPLEMENTARY FIGURES

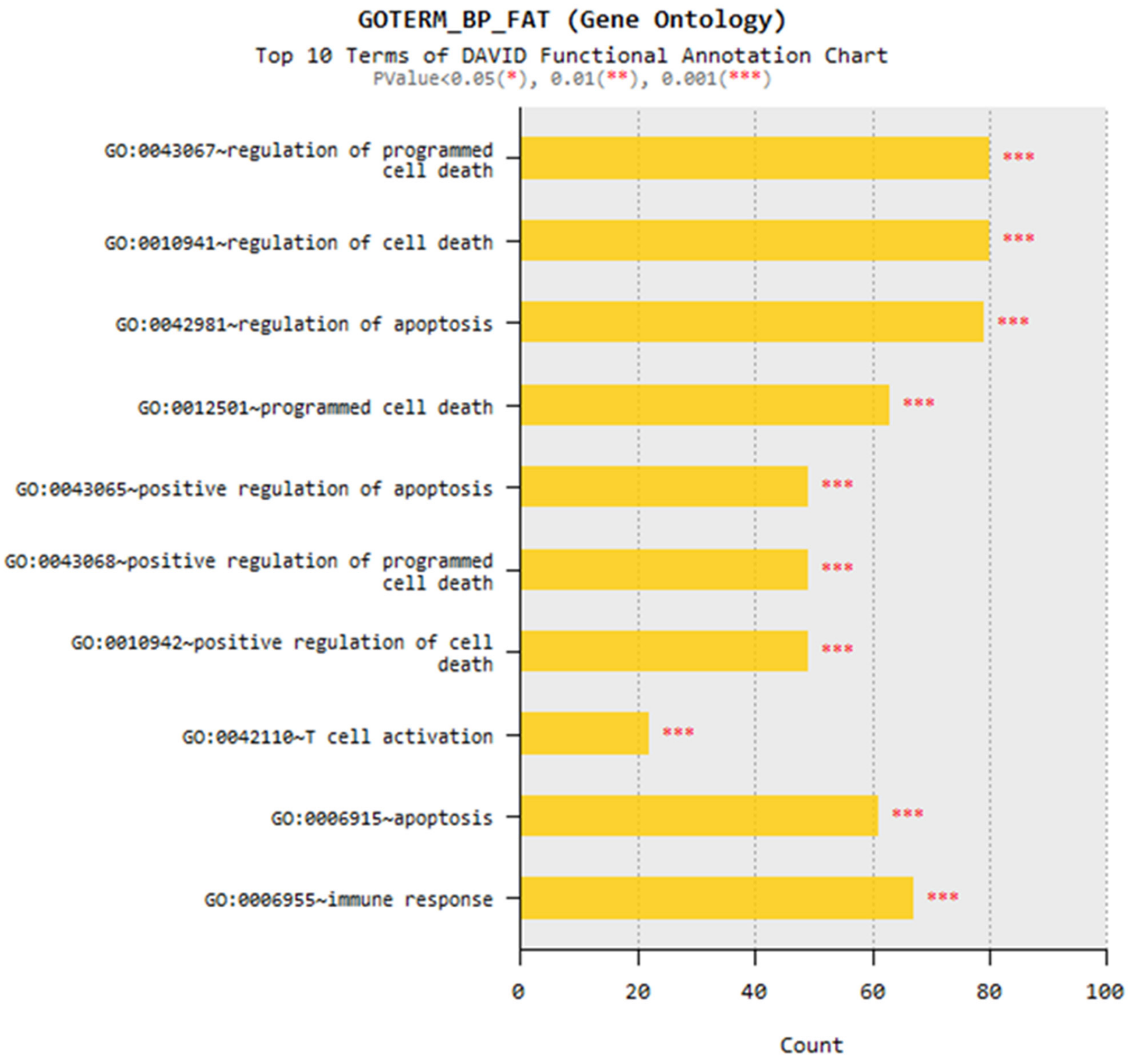


**Supplementary Figure S1: Genes related to biologic process were grouped into functional categories according to DAVID annotation chart.**

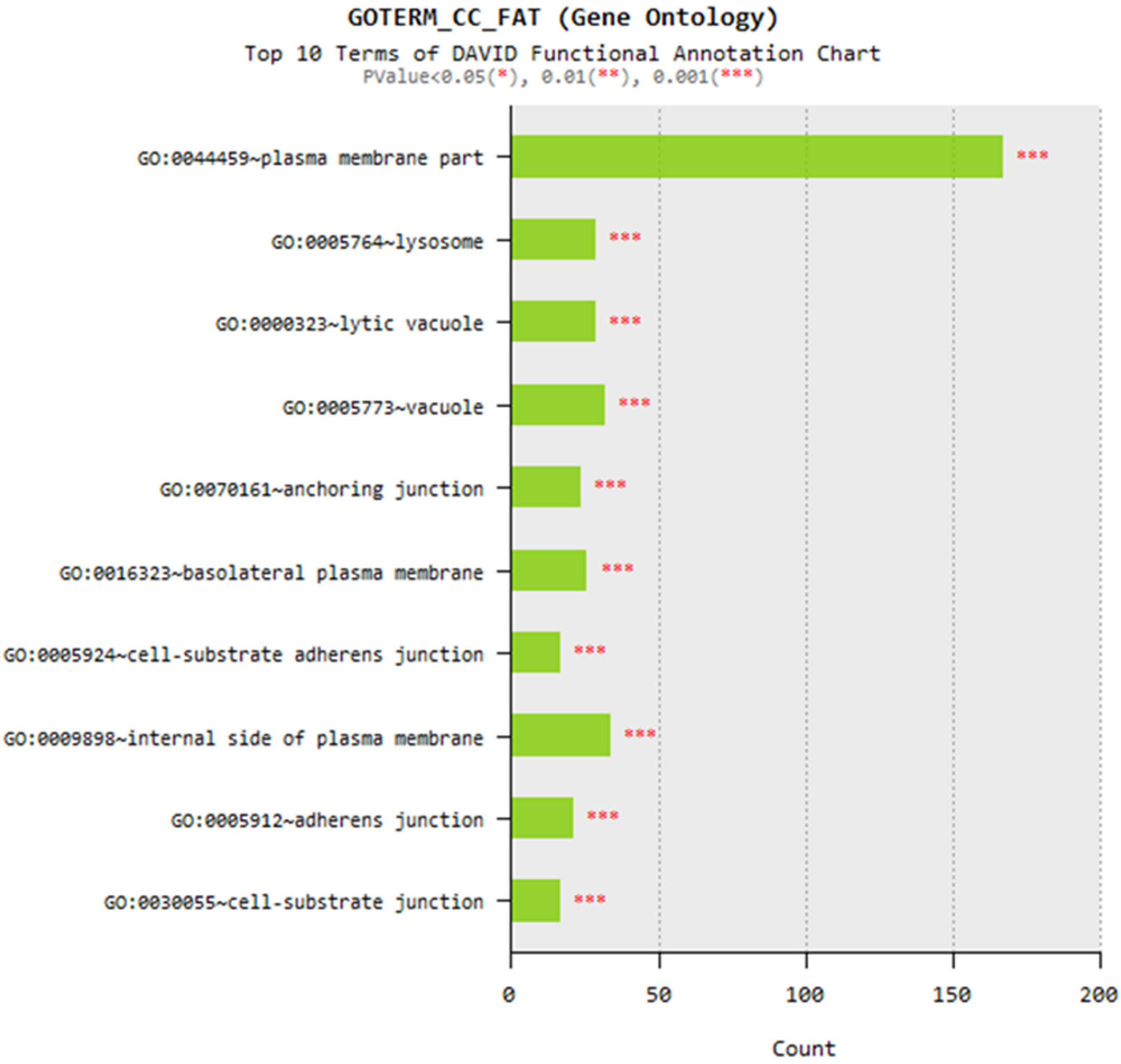


**Supplementary Figure S2: Genes related to cellular component were grouped into functional categories according to DAVID annotation chart.**

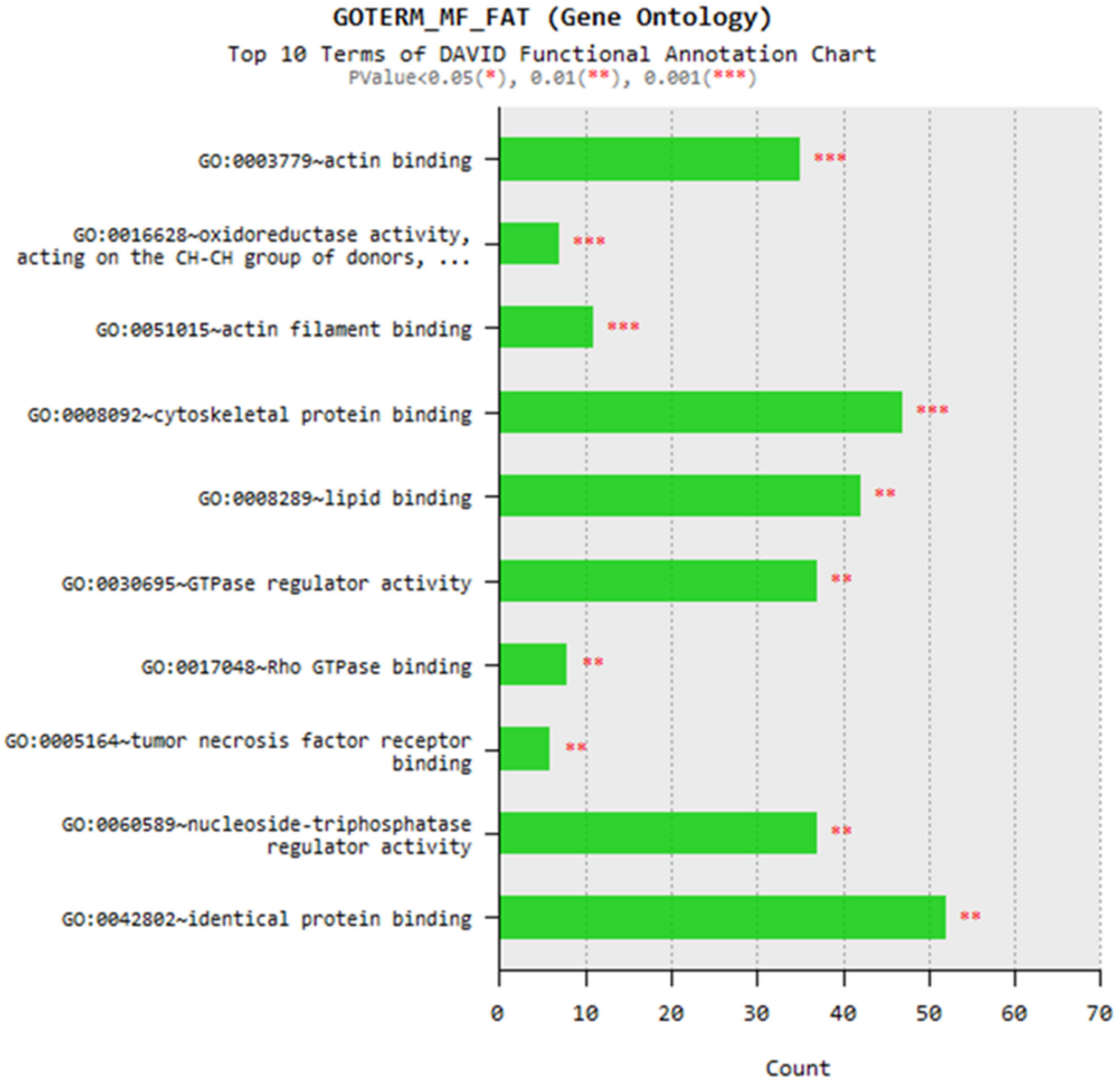


**Supplementary Figure S3: Genes related to molecular function were grouped into functional categories according to DAVID annotation chart.**
